# Effect and mechanism of chlorogenic acid on cognitive dysfunction in mice by lipopolysaccharide-induced neuroinflammation

**DOI:** 10.3389/fimmu.2023.1178188

**Published:** 2023-05-24

**Authors:** Siyuan Xiong, Xuyang Su, Yingjie Kang, Junqiang Si, Lu Wang, Xinzhi Li, Ketao Ma

**Affiliations:** ^1^ Key Laboratory of Xinjiang Endemic and Ethnic Diseases, Ministry of Education, Shihezi University School of Medicine, Shihezi, China; ^2^ National Health Commission (NHC) Key Laboratory of Prevention and Treatment of Central Asia High Incidence Diseases, First Affiliated Hospital, Shihezi University School of Medicine, Shihezi, China; ^3^ Department of Pathophysiology, Shihezi University School of Medicine, Shihezi, China; ^4^ Department of Physiology, Shihezi University School of Medicine, Shihezi, China; ^5^ Department of Pharmacology and Clinical Pharmacy, Shihezi University School of Pharmacy, Shihezi, China

**Keywords:** chlorogenic acid, neuroinflammation, cognitive dysfunction, microglia, polarization, TNF signaling pathway

## Abstract

**Background:**

Neuroinflammation is an important factor causing numerous neurodegenerative pathologies. Inflammation can lead to abnormal neuronal structure and function and even death, followed by cognitive dysfunction. There is growing evidence that chlorogenic acid has anti-inflammatory effects and immunomodulatory activity.

**Purpose:**

The aim of this study was to elucidate the potential targets and molecular mechanisms of chlorogenic acid in the treatment of neuroinflammation.

**Methods:**

We used the lipopolysaccharide-induced neuroinflammation mouse model and the lipopolysaccharide-stimulated BV-2 cells *in vitro* model. Behavioral scores and experiments were used to assess cognitive dysfunction in mice. HE staining and immunohistochemistry were used to assess neuronal damage in the mouse brain. Immunofluorescence detected microglia polarization in mouse brain. Western blot and flow cytometry detected the polarization of BV-2 cells. The migration of BV-2 cells was detected by wound healing assay and transwell assay. Potential targets for chlorogenic acid to exert protective effects were predicted by network pharmacology. These targets were then validated using molecular docking and experiments.

**Results:**

The results of *in vivo* experiments showed that chlorogenic acid had an obvious ameliorating effect on neuroinflammation-induced cognitive dysfunction. We found that chlorogenic acid was able to inhibit BV-2 cells M1 polarization and promote BV-2 cells M2 polarization *in vitro* while also inhibiting the abnormal migration of BV-2 cells. Based on the network pharmacology results, we identified the TNF signaling pathway as a key signaling pathway in which chlorogenic acid exerts anti-neuroinflammatory effects. Among them, Akt1, TNF, MMP9, PTGS2, MAPK1, MAPK14, and RELA are the core targets for chlorogenic acid to function.

**Conclusion:**

Chlorogenic acid can inhibit microglial polarization toward the M1 phenotype and improve neuroinflammation-induced cognitive dysfunction in mice by modulating these key targets in the TNF signaling pathway.

## 1 Introduction

Neuroinflammation, as an important pathological change in the development of neurodegenerative diseases (e.g., Alzheimer’s disease, Parkinson’s disease, amyotrophic lateral sclerosis), affects neuronal structure and function and can even lead to neuronal death (1, 2). Lipopolysaccharide (LPS) is a component of the outer wall of gram-negative bacterial cell walls and is expressed on the membrane surface of immune cells such as macrophages and microglia (3). Activation of TLR-4, a specific receptor for LPS, the results in the production of proinflammatory cytokines, oxidative stress factors and chemokines, all of which are key mediators in the development of neuroinflammation (4, 5). In previous studies, we found that LPS can cause cognitive dysfunction and a range of complex behaviors, including decreased learning memory, reduced motor complexity, increased anxiety, and the emergence of depressive behaviors (6). A previous study demonstrated that intraperitoneal injection of LPS can be used as a model to study neuroinflammation-induced cognitive dysfunction in mice (7). Microglia, as intrinsic immune cells of the mammalian central nervous system (CNS), play a key role in maintaining brain homeostasis by monitoring their surroundings under physiological conditions (8). Under pathological conditions, resting microglia are activated and polarized into two cellular phenotypes, M1 and M2, *via* the classical activation pathway and the alternative activation pathway, respectively; the M1 phenotype is proinflammatory, and the M2 phenotype is anti-inflammatory (9, 10). M1 microglia mainly produce proinflammatory cytokines such as TNF-α, interleukin-1 (IL-1β), interleukin-6 (IL-6), and oxidative stress factors such as nitric oxide (NO) and reactive oxygen species (ROS), causing damage to neurons in the surrounding environment (11). M2 microglia produce interleukin-4 (IL-4), interleukin-10 (IL-10) and other anti-inflammatory factors that antagonize the M1 pro-inflammatory response and exert a protective effect on neurons (11). M2 microglia play a crucial role in suppressing inflammation, scavenging toxic factors and protecting the brain (12). Therefore, altering the M1/M2 phenotype can influence the progression of inflammation, and promoting microglial polarization toward the M2 phenotype is a key target for the treatment of neuroinflammation-based diseases.

In recent years, Chinese herbal medicines have received wide attention for their safety and efficacy. We found that chlorogenic acid (CGA) is often present as an active ingredient in plants such as Eucommiae (Eucommia ulmoides Oliv.), Honeysuckle (Lonicera japonica Thunb.), and green coffee bean. There is growing evidence that CGA has multiple pharmacological effects, including antioxidant, antibacterial, antiviral, antitumor, and immunomodulatory effects (13). Based on the strong antioxidant and anti-inflammatory effects of CGA, many scholars have found that it has a good neuroprotective effect (14). In addition, it has been shown that CGA is able to cross the blood-brain barrier (BBB) and can treat certain neurological disorders (15). Several clinical and preclinical studies have shown that coffee extract (CGA, the main component) exhibits good therapeutic effects in Alzheimer’s disease and Parkinson’s disease (16, 17). Hermawati found that CGA improved memory loss and hippocampal cell death after transient total cerebral ischemia and prevented CA1 pyramidal cell death after bilateral common carotid artery occlusion (18). Therefore, CGA may have a potential therapeutic effect on cognitive dysfunction caused by neuroinflammation. However, it is unclear whether CGA can affect microglial polarization and thus exert a therapeutic effect. In the present study, we investigated the effect of CGA on microglial polarization in mice with cognitive dysfunction caused by neuroinflammation and explored the protective mechanisms of CGA. Our study found that chlorogenic acid was able to ameliorate neuroinflammation and associated cognitive dysfunction by inhibiting microglia activation. Furthermore, through network pharmacological analysis, we found that chlorogenic acid may act on the TNF signaling pathway. This study may help determine the translational application of chlorogenic acid in clinical treatment.

## 2 Materials and methods

### 2.1 Chemicals and reagents

BV-2 cells were purchased from the Cell Bank of the Chinese Academy of Sciences. Lipopolysaccharide (LPS) was purchased from Sigma−Aldrich (St. Louis, MO, USA). Chlorogenic acid was purchased from Shanghai Yuanye Biotechnology Co. Ltd. (Shanghai, China). Anti-iNOS (1:1000, ab178945) and anti-MMP9 (1:1000, ab228402) were purchased from Abcam. Anti-CD86 (1:1000, BM4121), anti-Arg-1 (1:1000, M01106-4), anti-IL-10 (1:1000, RP1015), anti-CD206 (1:1000, A02285-2), anti-CXCL12 (1:1000, BA1389), anti-CXCR4 (1:1000, A00031-4), anti-PTGS2 (1:1000, A00084), and anti-TNF (1:1000, BA0131) were purchased from Boster Biological Technology Co. Ltd. (Shanghai, China). Cleaved caspase-3 (1:500, GB11532), IBA-1 (1:500, GB12105), and CD206 (1:400, GB113497) were purchased from Wuhan Servicebio Biotechnology Co. Ltd. (Wuhan, China). p-Akt1 (1:1000, 9018S), Akt1 (1:1000, 75692S), p-NF-κB (1:1000, 3033S), NF-κB (1:1000, 8242S), p-ERK1/2 (1:1000, 4370T), ERK1/2 (1:1000, 4696S), p-P38 (1:1000, 4511T), and P38 (1:1000, 8690T) antibodies were purchased from CST.

### 2.2 Animals and experimental protocols

**Figure 1:**
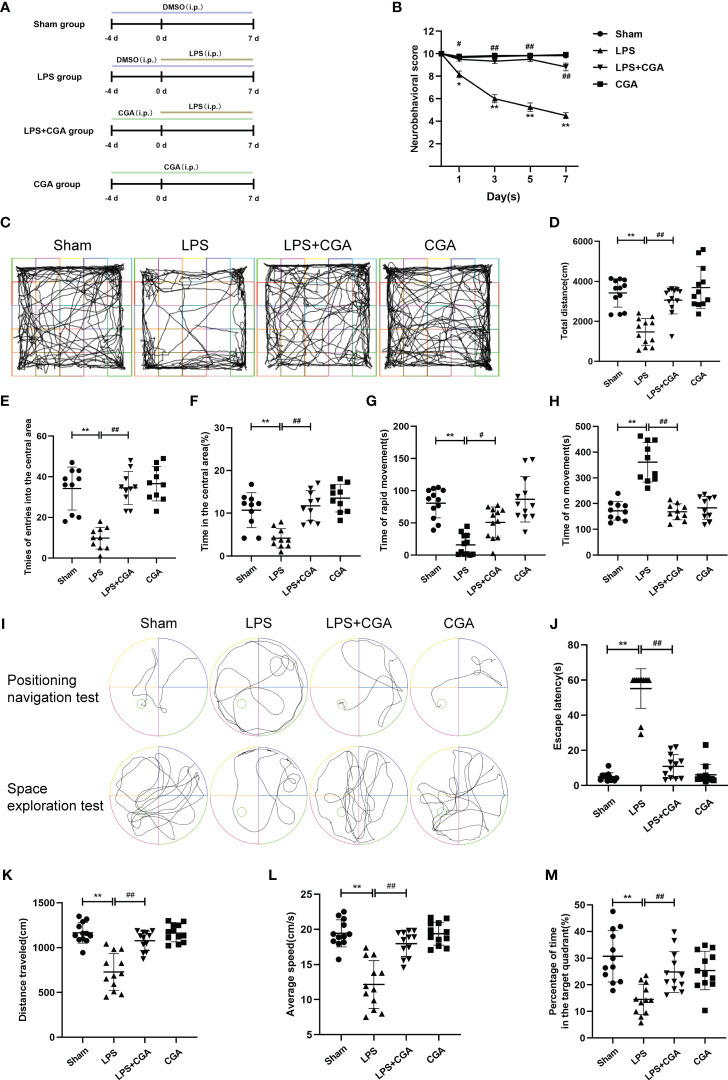
Chlorogenic acid improves cognitive dysfunction in a mouse model of LPS-induced neuroinflammation. **(A)** Timeline of *in vivo* experimental interventions. **(B)** Neurobehavioral scores at different time points. **(C-H)** Autonomous movement trajectory, total distance, number of times entering the central area, percentage of time in the central area and time of rapid movement and immobility of mice within ten minutes recorded in the open field experiment. **(I-M)** The Morris water maze test was used to record the autonomous movement trajectory map and escape latency of mice. The space exploration test recorded the autonomous movement trajectory, swimming distance, mean swimming speed and percentage of time in the target quadrant of mice. ^**^ *P* < 0.01 vs. the sham group, ^##^ *P* < 0.01 vs. the LPS group. (n=10).

Male C57BL/6 mice (18-22 g, 8-10 w) were obtained from Henan Speckles Biotechnology Co. Ltd. Housing conditions for all mice included a room temperature of 23°C, 50% humidity, and a 12-hour light-dark cycle. As shown in **Figure 1A**, 48 mice were randomly numbered and then randomly divided into four groups of 12 mice each: Sham group (DMSO-treated group), LPS group (lipopolysaccharide-treated group), LPS+CGA group (chlorogenic acid pretreatment + lipopolysaccharide-treated group), and CGA group (chlorogenic acid-treated group). The grouping of mice and the sample size of each group were determined based on previous studies(19, 20). The LPS+CGA group and the CGA group were injected intraperitoneally with CGA (40 mg·kg^−1^·d^−1^) daily for 11 d. The dose of chlorogenic acid is based on previous investigations and our pre-experiments (21). From the 5th day, LPS (750 μg·kg^-1^·d^-1^ (22)) was given intraperitoneally for another 7 days in the LPS and LPS+CGA groups, and the daily LPS injection time was second to the CGA injection for 3 h. In addition, DMSO (5%) was injected intraperitoneally daily in the Sham and LPS groups as a solvent control. Behavioral tests were performed on days 6-10, and mice were euthanized after 11 days by administering an overdose of sodium pentobarbital. The study protocol was approved by the Experimental Animal Ethics Committee of the First Affiliated Hospital of Shihezi University School of Medicine (licence number A2018-052-01).

### 2.3 Neurobehavioral score

The neurobehavioral score assesses four components: auricular reflex, flip-right reflex, corneal reflex, caudal flexion and escape response (23). A score of “0” means the mouse has no reflex, “1” means the mouse’s reflex time is more than 1 s, and “2” means the mouse’s reflex time is 1 s. The lower the score obtained by the mice, the more severe the damage to the nervous system of the mouse brain. Neurobehavioral scores were recorded and calculated by two independent and single-blind researchers.

### 2.4 Open field experiment

Three hours before the experiment, all mice were placed in the room for behavioral testing to adapt to the environment. A white experimental chamber with a size of 50 cm×50 cm×40 cm was placed for the mice to move freely in it. After setting the corresponding parameters in the software, the experimenter records the date, the number of animals and other information. Remove the mice from the cage, put them into the central area of the experimental box and leave them immediately. The trajectory of the mice in the experimental box for 10 min was monitored by the animal behavior analysis software, and the total distance the mice moved, the number of times they entered the central area, the percentage of time they were in the central area, and the percentage of time the mice moved at different speeds were recorded. The experimental chamber was cleaned with alcohol, and the next mouse was tested after an interval of 5-10 min.

### 2.5 Morris water maze test

Place a round platform in a round basin with a diameter of 1 m, and then add tap water of approximately 25°C to the basin, with the water surface 2-3 centimeters above the platform (22). Prior to LPS injection, we trained four groups of mice twice over a four-day period. Groups of mice were trained to find a hidden platform in the water maze within 60 s. If the mouse could not find the platform within 60 s, it was guided to the platform and allowed to stay on the platform for 10 s. The locus navigation experiment was performed on the fifth day after LPS injection, and the escape latency, i.e., the time from when the experimenter let go of the mouse to 3 s after the mouse remained on the platform, was recorded. On the next day, the platform was removed from the water maze, and the mice were subjected to spatial exploration experiments. The mice swam freely in the maze for 60 s, the trajectory of the mice was monitored, and data such as the average swimming speed, the distance swum and the percentage of total time spent in the target quadrant were recorded. The data were analyzed using Tracking Master V3.0 software (Shanghai Vanbee Intelligent Technology Co., Ltd.).

### 2.6 Tissue preparation and H&E staining

Mice were euthanized, perfused with saline *via* the heart, and then perfused with 4% paraformaldehyde solution. The brain tissue was carefully peeled out and fixed in 4% paraformaldehyde solution overnight. The tissue was dehydrated in ethanol and then embedded in paraffin to make 4 µm thick coronal sections of the brain. The paraffin sections were dewaxed in water, stained sequentially with hematoxylin and eosin, dehydrated and sealed, and neuronal damage in the hippocampal region of the mouse brain was observed by microscopy.

### 2.7 Immunohistochemistry

Prepared paraffin sections were dewaxed in water for antigen repair. The sections were covered uniformly with 3% BSA and blocked at room temperature for 30 min. Cleaved- caspase-3 primary antibody was added dropwise to the sections, which were incubated flat in a wet box at 4°C overnight. Then, the corresponding secondary antibody was incubated at room temperature. DAB color development solution was added dropwise, the color development time was controlled under the microscope, and the positive color was brownish yellow. Then, hematoxylin was restained for approximately 3 min, washed, and then hematoxylin differentiation solution was used to differentiate for a few seconds, washed, hematoxylin return blue solution returned to blue, and rinsed with running water. After dehydration and sealing, the sections were placed under a light microscope to observe neuronal damage in the hippocampal region of the mouse brain.

### 2.8 Immunohistofluorescence

Paraffin sections were dewaxed in water and then subjected to antigen repair. The sections were blocked with 3% BSA at room temperature. A mixed primary antibody of IBA-1 and CD86 or CD206 was added dropwise to the section and incubated overnight at 4°C, with the corresponding secondary antibody incubated at room temperature. DAPI was used to restain the cell nucleus. After washing, the slices were sealed. Sections were imaged with a fluorescence microscope, and pictures were collected to observe the polarization of microglia in the hippocampal region of the mouse brain.

### 2.9 Cell culture

BV-2 cells were cultured in MEM containing 10% serum, 0.5% penicillin and 0.5% streptomycin. The cell culture conditions were 37°C and 5% CO_2_. BV-2 cells were divided into the control group, the LPS group, and the LPS+CGA (50, 100, and 200 μM) group. After preintervention with CGA (50, 100, 200 μM) for 24 h, the cells were stimulated with LPS (1 μg·ml^-1^) for 24 h (24).

### 2.10 Western blotting analysis

Protein samples were electrophoresed on 10% or 12% SDS polyacrylamide gels (35-50 μg) and later transferred to PVDF membranes, which were blocked with 5% skim milk or 5% bovine serum albumin at room temperature for 2 h. Primary antibodies were added dropwise and incubated overnight at 4°C. The membranes were then incubated with the corresponding secondary antibodies at room temperature for 2 h. The PVDF membranes were incubated with enhanced chemiluminescence (ECL) reagents and exposed to a fully automated chemiluminescence image analysis system. The relative expression levels of target proteins were analyzed using ImageJ.

### 2.11 Flow cytometry assay

The treated BV-2 cells were transferred to centrifuge tubes, washed with PBS, counted, and blocked with 3% BSA for 30 min. The corresponding primary antibody was added and incubated at 37°C for 2 h. The corresponding fluorescent secondary antibody was added and incubated at 37°C for 2 h in the dark. After washing with PBS, the percentage of positive cells was detected by flow cytometry (BD, USA).

### 2.12 Wound healing assay

BV-2 cells were inoculated in 6-well plates and cultured until the cell density was approximately 90%. The cell layer was scratched with the tip of a 1 ml sterile pipette to form a straight line. After washing away floating cells and cell debris with PBS, interventions were performed in the above manner for different groupings. At 0 and 48 h after scratching, cell migration was observed by light microscopy, and images were collected. The number of migrating cells or the area of the trabeculae was counted using ImageJ.

### 2.13 Transwell assay

BV-2 cells (4×10^5^ cells·ml^-1^) were inoculated in the upper chamber of Transwell culture plates. The upper chamber was filled with MEM containing 2% serum, and the lower chamber was filled with MEM containing 10% serum. After intervention as described above, the plates were washed with PBS. After gently wiping off the unperforated cells in the upper chamber with a cotton swab, the perforated cells were fixed with 4% paraformaldehyde and stained with 0.1% crystal violet for 30 min. Four to five fields of view were randomly selected with a light microscope to count the number of perforated cells.

### 2.14 ELISA

The expression levels of TNF-α and IL-6 in mouse brain tissue homogenates were detected using ELISA kits. Within 5 min, the optical density values at 450 nm were detected by enzyme standardization, and the standard curve was plotted. The measurements were repeated three times for both standards and samples.

### 2.15 Network pharmacology analysis

In this study, the TCMSP database (http://tcmspw.com/tcmsp.php), SWISS target prediction database (http://swisstargetprediction.ch/), and QSAR database (Quantitative Structure Activity Relationship) were used to predict potential chlorogenic acid targets. The UniProt database was converted to UniPort ID (https://www.Unitprot.org/), and duplicate values were removed after merging the chlorogenic acid targets. The targets of neuroinflammation and cognitive dysfunction were obtained by searching “neuroinflammation” and “cognitive dysfunction”, and the databases included DisGeNET (https://www.disgenet.org/), G enecards database (https://www.genecards.org/), and OMIM database (https://www.omim.org/). The targets were converted to UniProt IDs using the UniProt database. Venn diagrams of common targets for chlorogenic acid, neuroinflammation and cognitive dysfunction were created using the OmicShare tool (https://www.omicshare.com/). The common targets were transformed into corresponding gene names using the UniProt database, and then protein interaction (PPI)-related information was obtained through the String database (https://string-db.org/). Visualization was performed, and the degree values of proteins and key proteins in the PPI network were obtained by Cytoscape 3.7.0 software.

### 2.16 Docking analysis

In this research, molecular docking was used to evaluate the interaction between chlorogenic acid and core targets. Three-dimensional (3D) crystal structures of the targets Akt1, TNF, MMP9, PTGS2, MAPK1, MAPK14, and RELA were obtained from the Protein Data Bank (PDB), and the two-dimensional (2D) SDF format of chlorogenic acid was downloaded from the PubChem database. The docking of chlorogenic acid to each target was performed using Autodock. The specific docking process is described as follows: First, the crystal structures were retrieved and downloaded from the PDB protein database. Secondly, the receptor macromolecule was deleted with ligands and excess water molecules as the receptor structure for molecular docking. Then the rotatable bonds of the small molecule drug are identified and set to add hydrogen atoms to the protein structure. Finally, the blind docking approach we used, the docking box was selected to dock the whole protein. In particular, the docking method we chose was semi-flexible docking with 100 docking times.

### 2.17 Statistical analysis

The experimental results were expressed as the mean ± standard deviation. Statistical analysis was performed using SPSS software using one-way ANOVA. P values <0.05 were considered statistically significant.

## 3 Results

### 3.1 Chlorogenic acid improved cognitive dysfunction in mice with LPS-induced neuroinflammation

We used a neurobehavioral scoring method at different periods to assess the effects of lipopolysaccharide-induced neuroinflammation on the brain. On the 3rd, 5th, and 7th days after LPS injection, the neurobehavioral scores of mice in the LPS group were lower than those in the Sham group, whereas the neurobehavioral scores of mice pretreated with chlorogenic acid were higher than those of mice in the LPS group (**Figure 1B**). Two days after LPS injection, we observed the voluntary locomotor ability of mice in each group by the open field experiment. Mouse activity trajectory plots showed that the locomotor complexity of mice in the LPS group was obviously reduced compared with that of mice in the sham group, and chlorogenic acid pretreatment was able to reverse the LPS-induced reduction in mouse activity complexity (**Figure 1C**). In addition, our data showed that the total distance moved, the number of times entering the central area, the percentage of time staying in the central area, and the duration of rapid locomotion were all reduced in the LPS group of mice compared to the Sham group, and pretreatment with chlorogenic acid improved these performances (**Figures 1D-H**). On the 6th and 7th days after LPS injection, we examined the learning and memory abilities of each group of mice by the Morris water maze test. The results of the positioning navigation test showed that the escape latency of mice in the LPS group was observably longer than that of mice in the sham group, while the escape latency of mice in the LPS+CGA group was shorter than that of mice in the LPS group (**Figures 1I, J**). The results of the space exploration test showed that LPS mice had lower swimming distance, average speed, and duration in the target quadrant than sham group mice, and chlorogenic acid-treated mice were able to attenuate the abovementioned performance caused by LPS (**Figures 1K-M**). These results suggest that chlorogenic acid can improve the cognitive dysfunction induced by LPS in mice.

### 3.2 Chlorogenic acid reduced the expression of LPS-induced inflammatory factors and attenuated neuronal damage in the mouse brain

**Figure 2:**
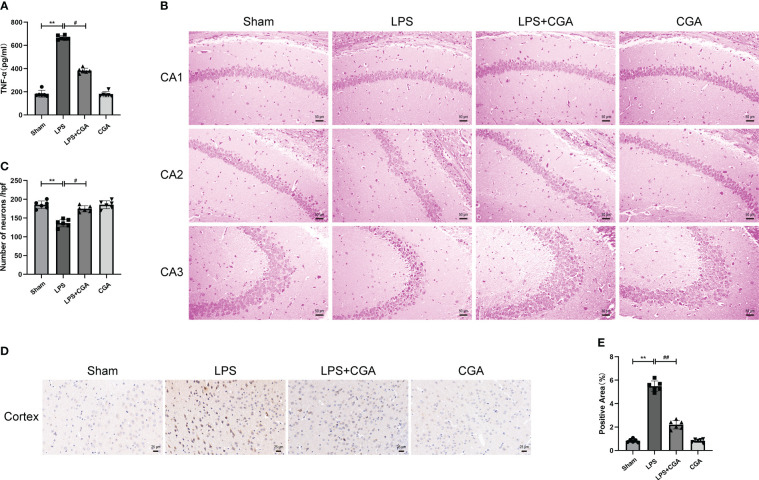
Chlorogenic acid pretreatment reduces the expression of inflammatory factors and neuronal damage in LPS-induced mouse brains. **(A)** Measurement of the expression levels of inflammatory factors in mouse brain tissue by ELISA. **(B, C)** HE staining of the hippocampus in different groups of mice. Magnification: ×200. **(D, E)** Immunohistochemical staining of cortical regions in different groups of mice. Magnification: ×400. ^**^ *P* < 0.01 vs. the sham group, ^#^ *P* < 0.05, ^##^ *P* < 0.01 vs. the LPS group. (n=6).

The expression of inflammatory factors in mouse brain tissue homogenates was measured by ELISA to assess the inflammatory response in the brain. The results showed that LPS induced increased levels of TNF-α secretion in brain tissue, while chlorogenic acid attenuated this inflammatory response (**Figure 2A**).

The normal survival of neurons in the brain is closely related to cognitive and memory functions in mice. To demonstrate the effect of chlorogenic acid on the survival of neurons in the mouse brain, we performed HE staining on brain tissue sections of mice. The results showed that neurons in the hippocampal region of the sham group were abundant and closely arranged, with normal neuronal morphology, clear nucleus-cytoplasm demarcation, obvious nucleoli, and no obvious pathological changes. The brain tissues of mice in the LPS group showed a decrease in the number of neurons in the CA1 region of the hippocampus, a disorganized arrangement of neurons in the CA2 region, deep staining of neuronal consolidation in the CA3 region, poorly delineated nuclei and cytoplasm, and increased basophilia. The neuronal damage in the brain tissues of mice pretreated with CGA was significantly improved (**Figures 2B, C**). We further examined the apoptosis of neurons in the mouse brains by immunohistochemistry. The results showed that there was an obvious increase in brownish-yellow particles in the cytoplasm of neuronal cells in the cerebral cortex of LPS mice compared with sham mice, while there was a decrease in brownish-yellow particles in the cytoplasm of neuronal cells in the cerebral cortex of LPS+CGA mice (**Figures 2D, E**). These results suggest that chlorogenic acid can improve LPS-induced neuronal damage in the mouse brain.

### 3.3 Chlorogenic acid regulated M1/M2 polarization levels in mouse hippocampal microglia

**Figure 3:**
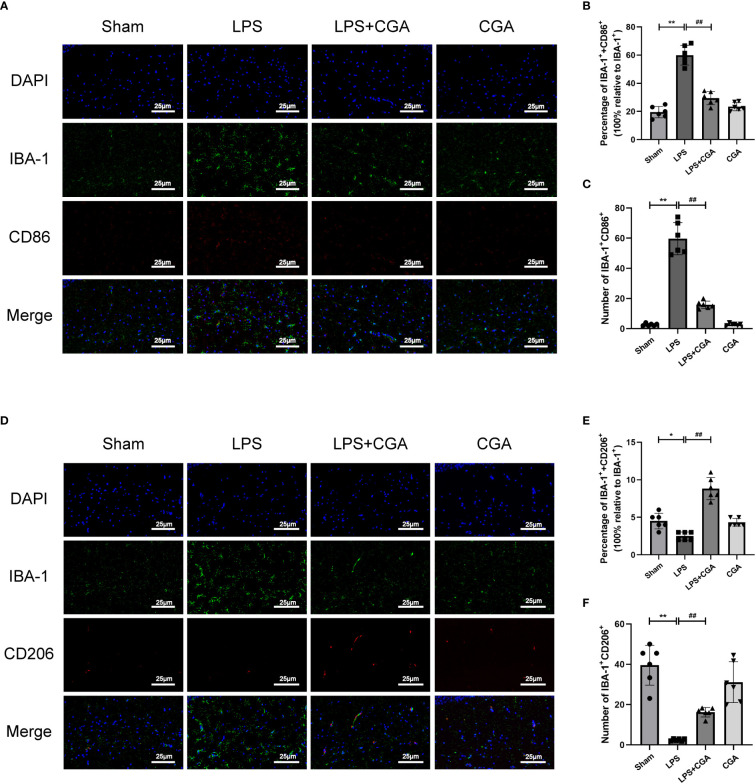
Chlorogenic acid inhibited the polarization of mouse hippocampal microglia toward M1 phenotype and promoted the polarization of microglia toward M2 phenotype. **(A-C)** Immunofluorescence staining of IBA-1/CD86 expression in microglia from different groups of mice. **(D-F)** Immunofluorescence staining of IBA-1/CD206 expression in microglia from different groups of mice.*P < 0.05, **P < 0.01 vs. the sham group, ##P < 0.01 vs. the LPS group. (n=6).

Microglia, as intrinsic immune cells in the brain, are inextricably linked to central nervous system inflammation. Abnormal activation or dysregulation of the polarization ratio of microglia can cause severe inflammatory responses in the brain. We analyzed the polarization levels of microglia in the brain by immunohistofluorescence. The results showed that IBA-1^+^ expression was increased in the brains of mice in the LPS group compared to the sham group, the expression of IBA-1^+^ and CD86^+^ was increased, and the expression of IBA-1^+^ and CD206^+^ was decreased. This indicates that LPS can induce microglial activation and polarization toward the M1 phenotype. Compared with the LPS group, the expression of IBA-1^+^ in the brains of mice in the LPS+CGA group was decreased, and the expression of IBA-1^+^ and CD86^+^ was decreased, while the expression of IBA-1^+^ and CD206^+^ was increased. The above results indicated that CGA pretreatment inhibited LPS-induced microglial activation, inhibited microglial polarization toward the M1 phenotype and promoted microglial polarization toward the M2 phenotype (**Figures 3A–F**).

**Figure 4:**
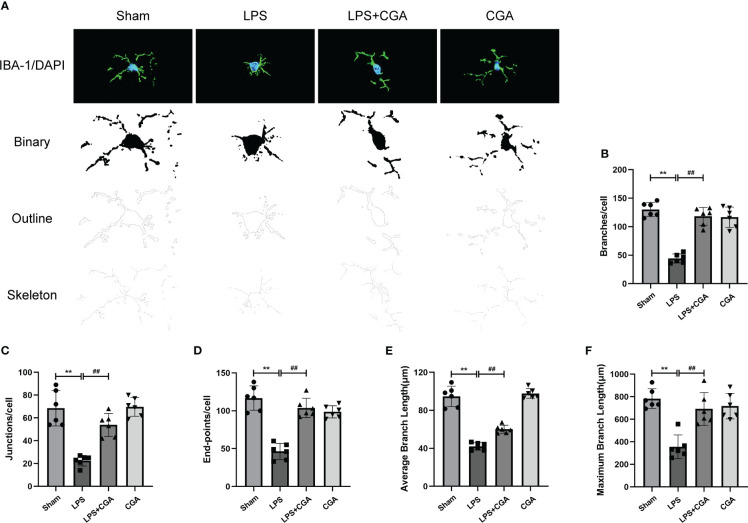
Chlorogenic acid reduces LPS-induced microglial activation in the hippocampal region of mice. **(A)** Binary, outline and skeleton images of microglia after Iba-1 labeling. **(B–F)** Statistical analysis of microglial cell branches, junctions, endpoints, average branch length, and maximum branch length. ^**^ *P* < 0.01 vs. the sham group, ^##^ *P* < 0.01 vs. the LPS group. (n = 6).

Next, we analyzed the morphology of microglia by Fiji, including the number of branches, junctions, endpoints, average branch length, and maximum branch length. The results showed that compared with the sham group, the microglia in the LPS group were in an activated state, the number of branches, junctions and endpoints of the cells were reduced, and their average branch length and maximum branch length were also decreased. Pretreatment with chlorogenic acid effectively inhibited microglial activation(**Figures 4A–F**).

### 3.4 Chlorogenic acid regulated M1/M2 polarization levels in BV-2 cells

**Figure 5:**
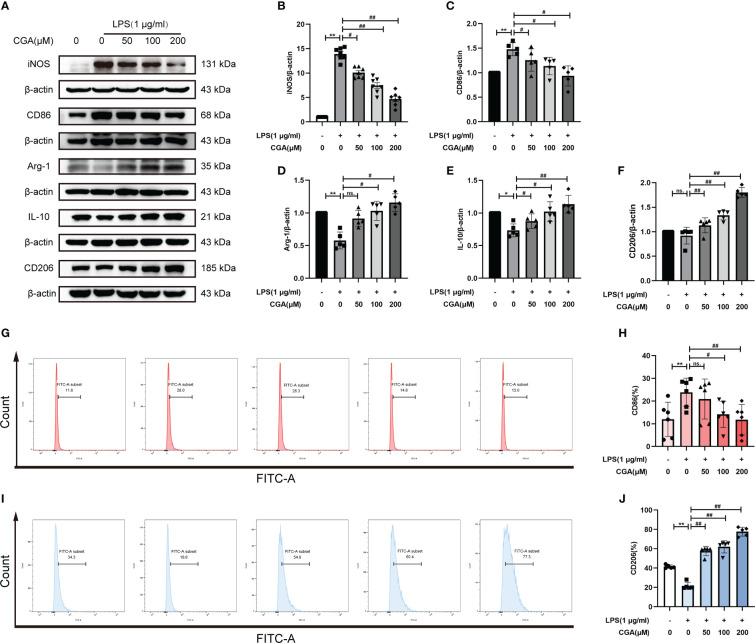
Chlorogenic acid regulates M1/M2 polarization levels in BV-2 cells. BV-2 cells were exposed to LPS (1 μg·ml^-1^) for 24 h with or without CGA pretreatment for 24 h. **(A–F)** Detection of the protein expression levels of iNOS, CD86, Arg-1, IL-10 and CD206 by Western blotting. **(G, H)** Detection of CD86 expression levels by flow cytometry. **(I, J)** Detection of CD206 expression levels by flow cytometry. ^**^ *P* < 0.01 vs. the control group, ^#^ *P* < 0.05, ^##^ *P* < 0.01 vs. the LPS group. (n=5).

To study the effect of CGA on the polarization level of BV-2 cells, we detected the expression of M1/M2 polarization-related proteins by Western blotting. The results showed that the expression of iNOS and CD86 was elevated in the LPS group compared with the control group, i.e., LPS stimulation polarized the cells toward the M1. While the expression of iNOS and CD86 was decreased after pretreatment with different concentrations of CGA (**Figures 5A–C**), the expression of Arg-1, IL-10 and CD206 was increased (**Figures 5A, D–F**). We further detected the expression of M1/M2 polarization markers by flow cytometry. Consistent with the Western blot results, the proportion of CD86^+^ cells was increased in the LPS group (**Figures 5G, H**), and the proportion of CD206^+^ cells was decreased (**Figures 5I, J**). Compared with the LPS group, the percentage of CD86^+^ cells decreased and the percentage of CD206^+^ cells increased after pretreatment with CGA. The above results indicated that chlorogenic acid could inhibit LPS-induced M1 polarization of microglia and promote M2 polarization of microglia.

### 3.5 Chlorogenic acid reduced the migration level of BV-2 cells

**Figure 6:**
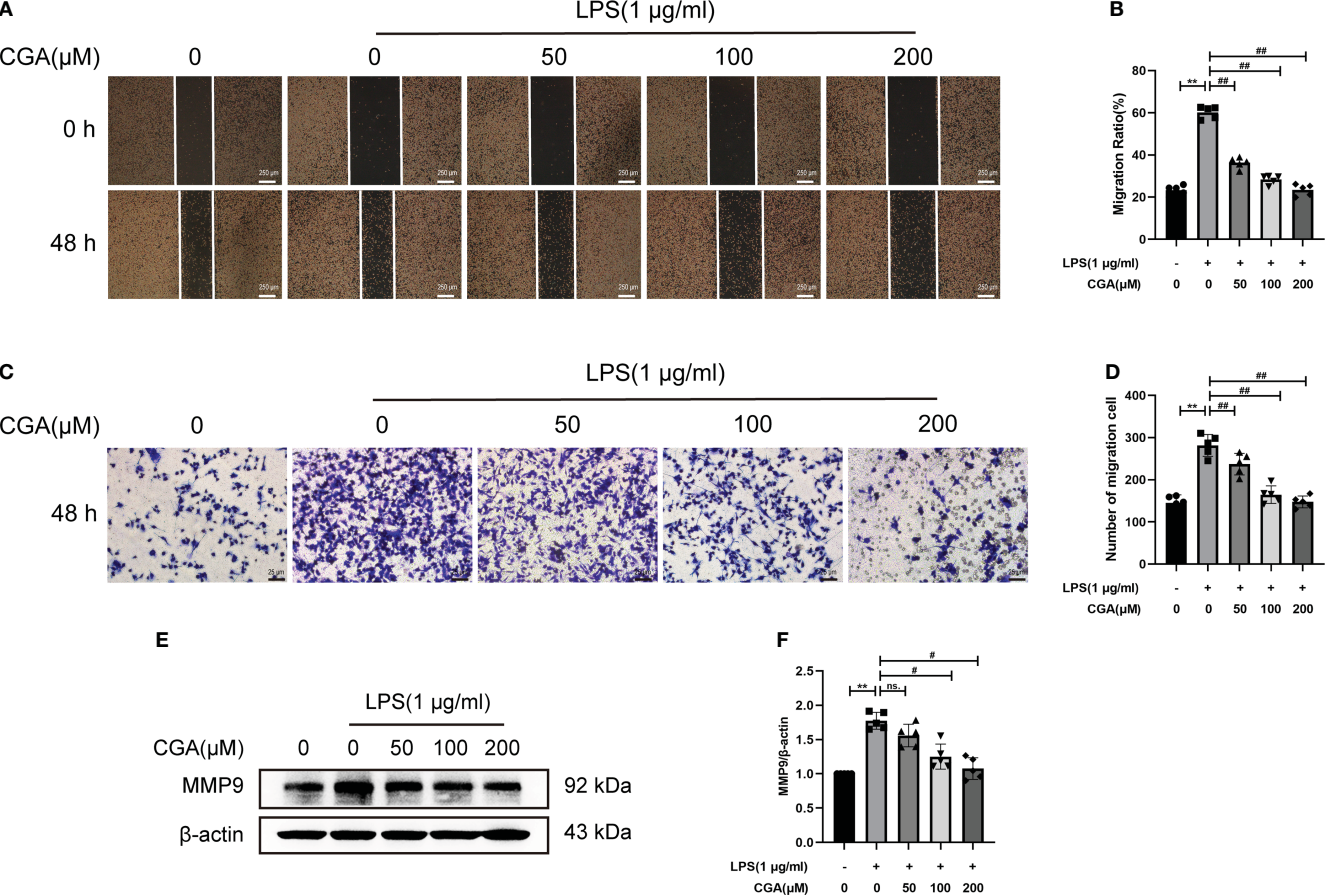
Chlorogenic acid inhibits the LPS-induced increase in the migration ability of BV-2 cells. BV-2 cells were exposed to LPS (1 μg·ml^-1^) for 24 h with or without CGA pretreatment for 24 h. **(A, B)** Wound healing assay of BV-2 cells migration at 48 h. **(C, D)** Transwell assay of BV-2 cells migration at 48 h. **(E, F)** Detection of MMP9 by Western blotting. ^**^ *P* < 0.01 vs. the control group, ^#^ *P* < 0.05, ^##^ *P* < 0.01 vs. the LPS group. (n=5).

Activated microglia are capable of producing large amounts of inflammatory mediators that cause damage to the surrounding environment. The increased migratory capacity of activated microglia leads to further amplification of the inflammatory response. To investigate the effect of CGA on the migratory capacity of BV-2 cells, we examined the lateral and longitudinal migration levels of BV-2 cells by scratch and Transwell assays. The results showed that LPS stimulation upregulated the lateral and longitudinal migration levels of BV-2 cells, while CGA pretreatment effectively attenuated the migration ability of BV-2 cells (**Figures 6A–D**). We further examined the expression of migration-associated proteins by Western blotting. The results showed that LPS induced the upregulation of MMP9 expression, while CGA pretreatment was able to reduce the expression of MMP9 (**Figures 6E, F**). All these results indicated that microglial migration levels were increased in the inflammatory environment and that CGA was able to inhibit microglial migration.

### 3.6 Chlorogenic acid improved neuroinflammation-induced cognitive dysfunction *via* the TNF signaling pathway

**Figure 7:**
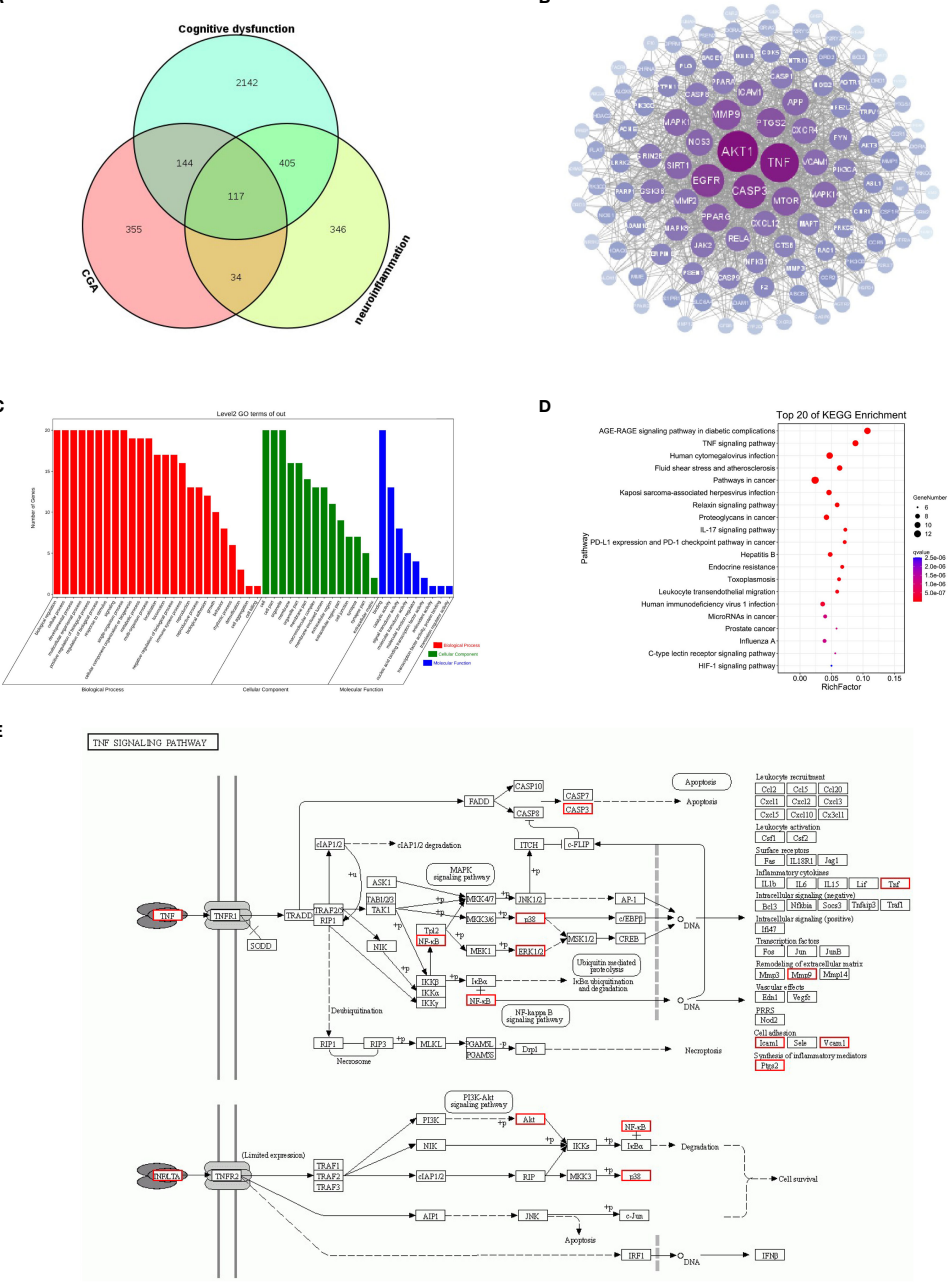
Results of network pharmacology analysis. **(A)** Cognitive dysfunction targets and neuroinflammation targets were obtained from the GeneCards database, and chlorogenic acid targets were obtained from the TCMSP database, Swiss target prediction database and QSAR database. A total of 117 targets were obtained after intersection of the Venn diagram. **(B)** PPI was constructed using 117 targets based on the degree values. **(C)** GO enrichment analysis of 20 targets. **(D)** KEGG pathway analysis of 20 targets. **(E)** Schematic diagram of TNF signaling pathway.

To further investigate the potential mechanisms by which CGA improves neuroinflammation-induced cognitive dysfunction, we analyzed this using web-based pharmacology-related databases. A total of 650 chlorogenic acid-related targets were obtained from the TCMSP database, Swiss target prediction database, and TargetNet database. A total of 902 targets related to neuroinflammation and 650 targets related to cognitive dysfunction were obtained from the GeneCards and OMIM databases, and 117 common targets for chlorogenic acid, neuroinflammation and cognitive dysfunction were identified (**Figure 7A**). The PPI network of these 117 potential targets was constructed from the String database. The 117 targets were visualized using Cytoscape 3.8.0 according to the degree value in descending order (**Figure 7B**). The top 20 proteins were Akt1, TNF, CASP3, EGFR, MMP9, PTGS2, MTOR, PPARG, SIRT1, MAPK1, NOS3, APP, ICAM1, CXCR4, MAPK14, VCAM1, CXCL12, RELA, MMP2, and JAK.

We further performed GO and KEGG enrichment analyses on the top 20 targets. The results of GO enrichment analysis suggested that the top 20 targets were mainly involved in immune system regulation and intracellular signaling (**Figure 7C**). The results of KEGG enrichment analysis showed that the top 20 targets were enriched in the AGE-RAGE signaling pathway, TNF signaling pathway, relaxin signaling pathway, and IL-17 signaling pathways, among which the TNF signaling pathway was closely related to inflammation (**Figure 7D**). The above results suggest that the TNF signaling pathway may be a key pathway by which CGA improves cognitive dysfunction caused by neuroinflammation (**Figure 7E**). Moreover, Akt1, TNF, MMP9, PTGS2, MAPK1, MAPK14, and RELA are closely related to the development of inflammation, and they may become key targets for chlorogenic acid to exert its protective effects.

### 3.7 Chlorogenic acid inhibited LPS-induced activation of Akt1, TNF, MMP9, PTGS2, MAPK1, MAPK14, and RELA targets in the TNF signaling pathway

**Figure 8:**
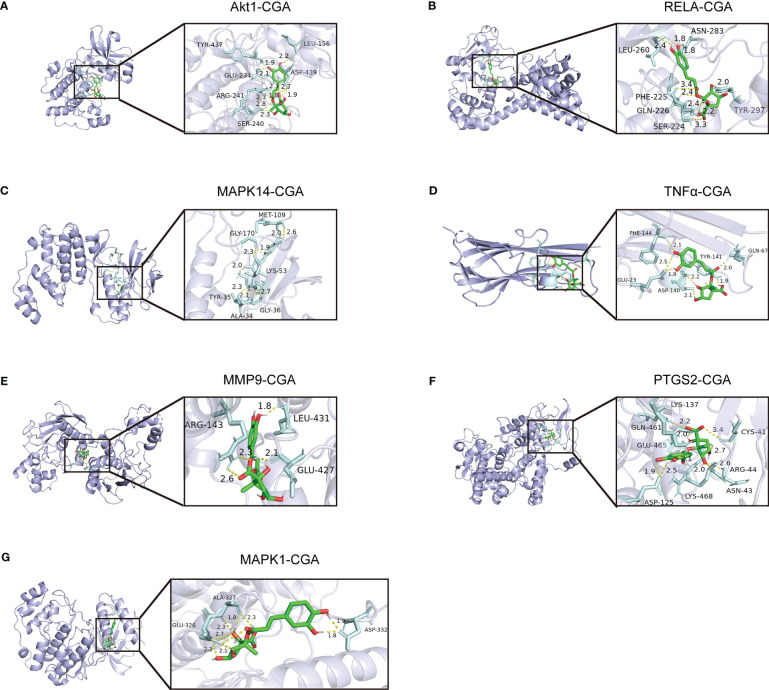
Molecular mechanisms by which chlorogenic acid improves neuroinflammation-induced cognitive dysfunction. **(A)** Three-dimensional docking of CGA and Akt1. **(B)** Three-dimensional docking of CGA and RELA. **(C)** Three-dimensional docking of CGA and MAPK14. **(D)** Three-dimensional docking of CGA and TNFα. **(E)** Three-dimensional docking of CGA and MMP9. **(F)** Three-dimensional docking of CGA and PTGS2. **(G)** Three-dimensional docking of CGA and MAPK1.

We evaluated the interaction between chlorogenic acid and Akt1, TNF, MMP9, PTGS2, MAPK1, MAPK14, and RELA by molecular docking. CGA docked with Akt1, RELA, MAPK14, MAPK1, TNFα, MMP9, and PTGS2 (**Figures 8A–G**). A small molecule-protein docking fraction <-1.2 indicates good binding between the two, so chlorogenic acid binds well to the above target molecules in docking and corroborates that chlorogenic acid may act through the above targets (**Table 1**).

**Table 1:** Molecular docking binding of chlorogenic acid to different targets.

Target	PDB	Score (Kcal/mol)
Akt1	3MVH	-6.96
RELA	3rc0	-5.45
MAPK14	6hwt	-6.99
MAPK1	6g54	-6.55
TNFα	6x86	-5.56
MMP9	5th6	-7.33
PTGS2	5IKT	-6.61

**Figure 9:**
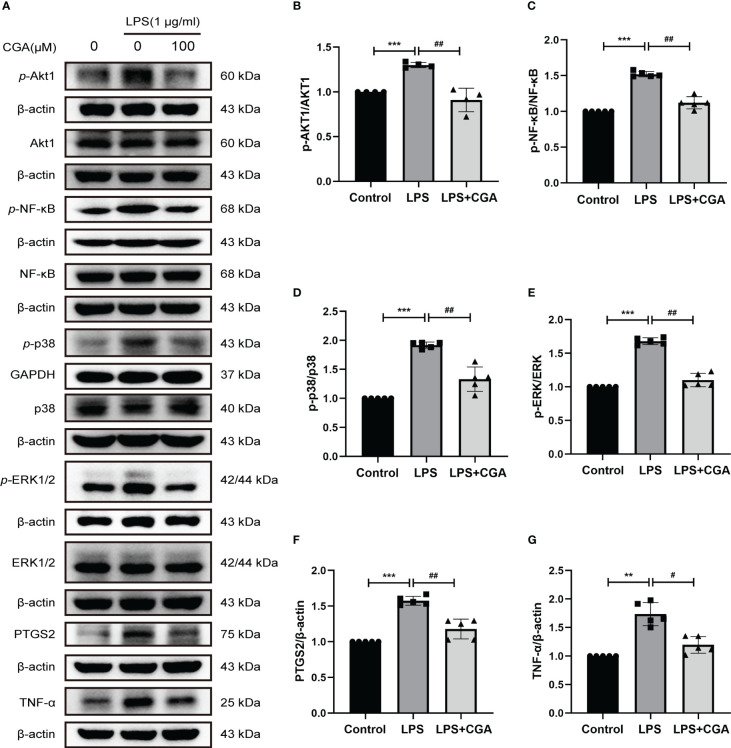
Chlorogenic acid downregulated the expression of core targets in the TNF signaling pathway. BV-2 cells were exposed to LPS (1 μg·ml^-1^) for 24 h with or without CGA pretreatment for 24 h. **(A–G)** Western blotting analysis of the protein expression of p-Akt1, Akt1, p-NF-κB, NF-κB, p-p38, p38, p-ERK1/2, ERK1/2, PTGS2, and TNF-α. **P < 0.01, ***P < 0.001 vs. the control group, #P < 0.05, ##P < 0.01 vs. the LPS group.(n=5).

Then, we further examined the effects of chlorogenic acid on Akt1, TNF, MMP9, PTGS2, MAPK1, MAPK14, and RELA targets by Western blotting. The results revealed that LPS stimulation increased the protein expression levels of p-Akt1, p-NF-κB, p-p38, p-ERK1/2, MMP9, PTGS2, and TNFα in BV-2 cells, and pretreatment with chlorogenic acid inhibited the upregulation of these proteins (**Figures 9A–G**, **Figures 6E, F**). The above results indicated that chlorogenic acid was able to inhibit LPS-induced activation of Akt1, TNF, MMP9, PTGS2, MAPK1, MAPK14, and RELA targets in the TNF signaling pathway.

## 4 Discussion

Neuroinflammation is an important feature in the pathogenesis and progression of many neurodegenerative diseases, and inhibition of neuroinflammation can be a key target for the treatment of neurodegenerative diseases (25). The establishment of neuroinflammation models in mice facilitates the screening of drugs with ameliorating effects on neuroinflammation. Chlorogenic acid, found in a variety of plants, has been shown to have anti-inflammatory effects. However, its specific role in microglial cell polarization in neuroinflammation has rarely been reported. The aim of this study was to investigate the effects and mechanisms of chlorogenic acid on mouse microglia and neurons during neuroinflammation. Previously, it has been shown that either intraperitoneal or intracerebral injection of LPS leads to the production of inflammatory factors in the brains of mice (26, 27). Our results likewise showed that intraperitoneal injection of LPS resulted in elevated expression of inflammatory factors in mouse brain tissue homogenates, whereas chlorogenic acid decreased the expression of inflammatory factors in the mouse brain, indicating that chlorogenic acid was able to inhibit LPS-induced neuroinflammation. Neuroinflammation is not only reflected in elevated expression of inflammatory factors alone but also leads to a series of changes in the brain, such as neuronal degeneration and death an microglia activation (28). Gao suggested that LPS-induced neuroinflammation leads to an increase in the number of damaged neurons in the CA1, CA2, CA3 and DG regions of the hippocampus in mice (29). Wang proposed that immunofluorescence results in cortical areas 6 s after LPS intracerebral injection showed reduced NeuN expression levels, indicating a decrease in the number of cortical neurons in mice (30). Similarly, we also observed neurons in the brain. The results showed that neurons in both the hippocampal and cortical regions of mice in the LPS group showed different degrees of damage and apoptosis, while chlorogenic acid-pretreated mice were protected from neuronal damage caused by LPS.

Abnormal neuronal function and death can lead to abnormal behavior in mice. Previous studies have shown that intraperitoneal injection of LPS leads to the production of IFN-γ and TNF-α in the mouse brain, and these two inflammatory factors have been shown to play an important role in LPS-induced depression-like behavior (31). The sucrose preference test in this study showed the same results. In addition, the results of Morri’s water maze in a previous study showed that LPS causes deficits in learning and memory function in animals (22, 32). Our results showed that mice subjected to LPS stimulation had reduced autonomic activity. The water maze experiment showed that the learning memory ability of mice was decreased after being subjected to LPS. All of the above behavioral changes in mice triggered by LPS were improved in the LPS+CGA group mice.

Microglia are a class of intrinsic immune cells found in the central nervous system and are involved in immune surveillance, signaling, injury response, phagocytosis of cellular debris and repair of synapses (33, 34). Since microglia are a major source of inflammatory factors, their overactivation leads to neuroinflammation, which is key to the development of many neurological diseases (35, 36). Activated microglia are generally divided into two phenotypes, M1 and M2, which exert proinflammatory and anti-inflammatory effects, respectively. Cytokines and chemokines released from M1 microglia induce the release of inflammatory factors and cytotoxic substances from leukocytes and macrophages, mediating neuroinflammation and neurotoxicity, leading to blood−brain barrier disruption and glial cell death (37); M2 microglia secrete anti-inflammatory factors such as IL⁃10 and IL⁃4 and neurotrophic factors such as transforming growth factor ⁃β (TGF⁃β), insulin growth factor (IGF) and vascular endothelial growth factor (VEGF), which play a role in reducing inflammation and restoring homeostasis in the body (38). Therefore, an increasing number of studies are targeting the microglial phenotype to inhibit neuroinflammation to improve or reverse the pathological process of neurological diseases (39, 40). In this study, double immunofluorescence staining of IBA-1 with CD86/CD206 showed that chlorogenic acid inhibited LPS-induced M1 polarization and promoted M2 polarization of microglia in the hippocampal region of mice. The results of *in vitro* experiments showed that chlorogenic acid could inhibit LPS-induced M1 polarization in BV-2 cells and promote M2 polarization in microglia. The above results suggest that chlorogenic acid can ameliorate LPS-induced neuroinflammation by regulating microglial polarization. Previously, the role of chlorogenic acid in microglial polarization has not been reported, and our study provides new ideas and evidence for chlorogenic acid in the treatment of neuroinflammation.

Network pharmacology is a novel research method for identifying putative targets and pharmacological mechanisms (41). To investigate the mechanism by which chlorogenic acid improves neuroinflammation-induced cognitive dysfunction, we used network pharmacology for further analysis. A total of 117 targets were found for chlorogenic acid, neuroinflammation and cognitive dysfunction, and GO and KEGG analyses were performed on the top 20 targets ranked by degree value. KEGG enrichment analysis showed a high enrichment score for the TNF signaling pathway. The targets enriched in the TNF signaling pathway were Akt1, TNF, MMP9, PTGS2, MAPK1, MAPK14, and RELA. Akt1 was the first target ranked by degree value in the network pharmacology results, suggesting that Akt1 may be an important target for chlorogenic acid action in neuroinflammation. Akt is an important signaling molecule that regulates many cellular processes, such as cell growth, survival and metabolism (42). Inflammation-associated proteins are often located downstream of Akt, which plays an important role in the development of inflammation. Inactivation of the JNK/Akt/NF-κB signaling pathway inhibits microglia-mediated inflammation in mice with experimental autoimmune encephalomyelitis (43). Ethanol and LPS were able to induce liver injury and activate the Akt signaling pathway with elevated expression of three Akt isoform phosphorylations (44). Our data show that the level of Akt1 phosphorylation is increased in activated microglia and that chlorogenic acid is able to reduce p-Akt1 expression.

Previous studies have shown that microglial activation is closely associated with the MAPK pathway. MAPK belongs to a family of highly conserved serine/threonine protein kinases, consisting of extracellular signal-regulated kinase (ERK), c-Jun N-terminal kinase (JNK)/stress-activated protein kinase, and p38 MAPK (45). Among them, ERK is the signal transduction protein that transmits mitogenic signals, and ERK1/2 is the most classical. Treatment of lipopolysaccharide-activated BV-2 cells with the ERK inhibitor SCH772984 resulted in an inhibition of NO release and a decrease in phosphorylated ERK (46). p38 MAPK is a stress-activated protein kinase. Activated p38 MAPK activates protein kinases, cytosolic proteins, cytoplasmic proteins, and transcription factors, which are involved in cell differentiation, apoptosis, senescence, inflammatory responses, and cytokine production (45, 47, 48). One study found that phosphorylated p38 MAPK was predominantly expressed in activated spinal microglia 1 d after surgery for lumbar disc herniation in rats (49). Our results indicate that chlorogenic acid suppresses neuroinflammation by inhibiting the phosphorylation of ERK1/2 and P38. In addition, microglial activation is closely related to the NF-κB pathway. NF-κB consists of five members, RElA (p65), RElB, c-REl, NF-κB1 (p50) and NF-κB2 (p52) (50). BV-2 cells activated by lipopolysaccharide *in vitro* showed increased release of inflammatory factors such as TNF-α and ROS-like, increased levels of phosphorylated NF-κB, and promoted p65 nuclear translocation (51, 52). Blocking the NF-κB pathway inhibits neurotoxin secretion, inflammatory factor release, and microglial activation, reducing the neuroinflammatory response. Our work showed that chlorogenic acid inhibits the release of the inflammatory factor TNF-α by inhibiting the phosphorylation of NF-κB.

In addition, the PTGS2 and MMP9 targets in the network pharmacology results are equally relevant to neuroinflammation. Previous research suggested that inhibition of mTOR can reduce microglial proliferation and regulate microglial activation by reducing the expression of iNOS and COX-2 in inflammatory cytokines (53). Therefore, PTGS2 may be a key therapeutic target for inflammation-mediated neurological disorders. Our results are consistent with previous reports that chlorogenic acid inhibited inflammation-mediated PTGS2 production.

MMP9 belongs to the matrix metalloprotein (MMP) family, whose main function is to degrade and remodel the extracellular matrix. Lin proposed that intracerebral injection of LPS increased MMP9 expression in the mouse brain (26). Overproduction of MMP9 leads to disruption of the blood−brain barrier and death-inducing ligand release, propagating neuroinflammatory responses through the recruitment of immune cells. Inhibition of MMP9 has been shown to protect against neuronal death and improve neurological function (54, 55). Our findings are consistent with a previous study showing that LPS promotes increased levels of microglial MMP9 expression, which increases the migration level of microglia and causes an increased inflammatory response. Moreover, wound healing and transwell assays further corroborated the change in the migration level of BV-2 cells after stimulation by LPS. In contrast, chlorogenic acid treatment inhibited the increased level of microglial migration in the inflammatory environment, inhibiting further amplification of inflammation to some extent.

In summary, chlorogenic acid exerted anti-inflammatory effects by inhibiting the activation of Akt1, NF-κB, ERK1/2, and p38 in the TNF signaling pathway and suppressing the expression of MMP9, PTGS2, and TNF-α. Our study provides new ideas for the treatment of neuroinflammation-induced cognitive dysfunction and strong evidence for the specific mechanism by which chlorogenic acid exerts its anti-inflammatory effects.

## 5 Conclusion

In summary, this study used network pharmacology analysis, molecular docking techniques, and *in vivo* and *in vitro* experiments to elucidate the role played by chlorogenic acid in a model of LPS-induced neuroinflammation and to explore the potential mechanisms by which chlorogenic acid acts. Our results showed that chlorogenic acid inhibited LPS-induced microglia activation and M1 phenotype polarization, promoted microglia polarization to M2 phenotype, reduced the release of inflammatory factor TNF-α, and decreased the degree of neuronal damage in hippocampal and cortical regions, thereby improving neuroinflammation as well as neuroinflammation-induced cognitive dysfunction in mice.Network pharmacology results suggest that the mechanism by which chlorogenic acid ameliorates neuroinflammation-induced cognitive dysfunction mainly involves the TNF signaling pathway. Molecular docking and *in vitro* experimental results show that the specific mechanism by which chlorogenic acid exerts its protective effect is through the inhibition of the activation of NF-κB and MAPK signaling in the TNF signaling pathway and the inhibition of the expression of inflammatory mediators TNF-α, MMP9, and PTGS2.Although the validation of the mechanism is not fully in-depth, our findings provide a partial research basis for the application of chlorogenic acid and offer new ideas for clinical prevention and treatment of neuroinflammation-induced cognitive dysfunction.

## Data availability statement

The original contributions presented in the study are included in the article/**Supplementary Material**. Further inquiries can be directed to the corresponding authors.

## Ethics statement

The animal study was reviewed and approved by Experimental Animal Ethics Committee of the First Affiliated Hospital, School of Medicine, Shihezi University (license number A2018-052-01).

## Author contributions

SX completed the experiments, wrote and charted the paper; XS wrote and revised the paper; YK assisted with mouse modeling and behavioral assays; JS completed data analysis and article revision; LW completed the network pharmacology analysis and molecular docking; XL and KM guided the research and revised the paper. All authors contributed to the article and approved the submitted version.
