# Supplementary material for: Effect and mechanism of chlorogenic acid on cognitive dysfunction in mice by lipopolysaccharide-induced neuroinflammation
Source: Front Immunol. 2023 May 24;14:1178188. doi: 10.3389/fimmu.2023.1178188 (PMC10244504; doi:10.3389/fimmu.2023.1178188)
Supplement: Supplementary file 1 [file DataSheet_1.docx]

Supplementary Material

**Effect and mechanism of chlorogenic acid on cognitive dysfunction in mice due to lipopolysaccharide-induced neuroinflammation**

**Siyuan Xiong ^1,2,3†^ , Xuyang Su^1,2,3†^, Yingjie Kang^1,2,4^, Junqiang Si^1,2,4^, Lu Wang^1,2,5*^, Xinzhi Li^1,2,3*^, Ketao Ma^1,2,4*^**

*** Correspondence:**

Ketao Ma, [maketao@hotmail.com](mailto:maketao@hotmail.com); Xinzhi Li, [lixinzhi@shzu.edu.cn](mailto:lixinzhi@shzu.edu.cn); Lu Wang, [1372584508@qq.com](mailto:1372584508@qq.com).

**^†^**These authors contributed equally to this work.

We have 2 supplementary figures as follows.


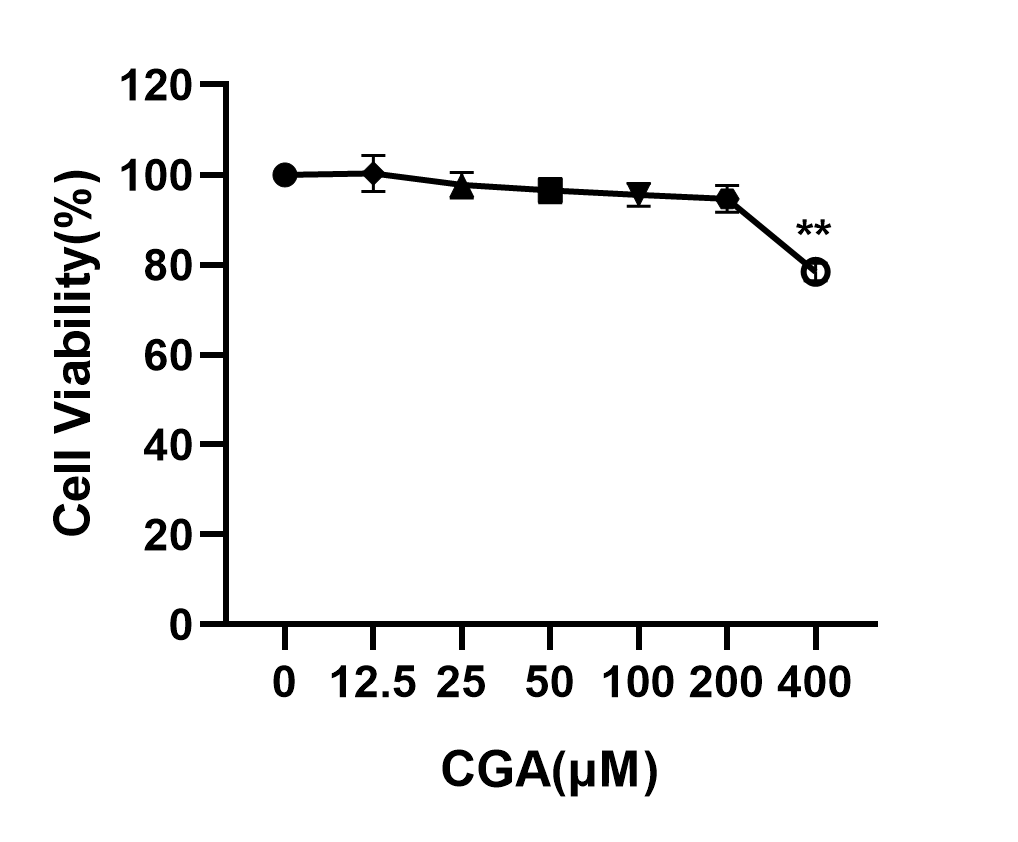


**Supplementary Figure 1.** Effect of different concentrations of chlorogenic acid on the survival rate of BV-2 cells. Intervention of BV-2 cells with 0,12.5,25, 50,100,200 μM chlorogenic acid for 24 hours and assay of cell viability.***P* < 0.01 vs. the control group.


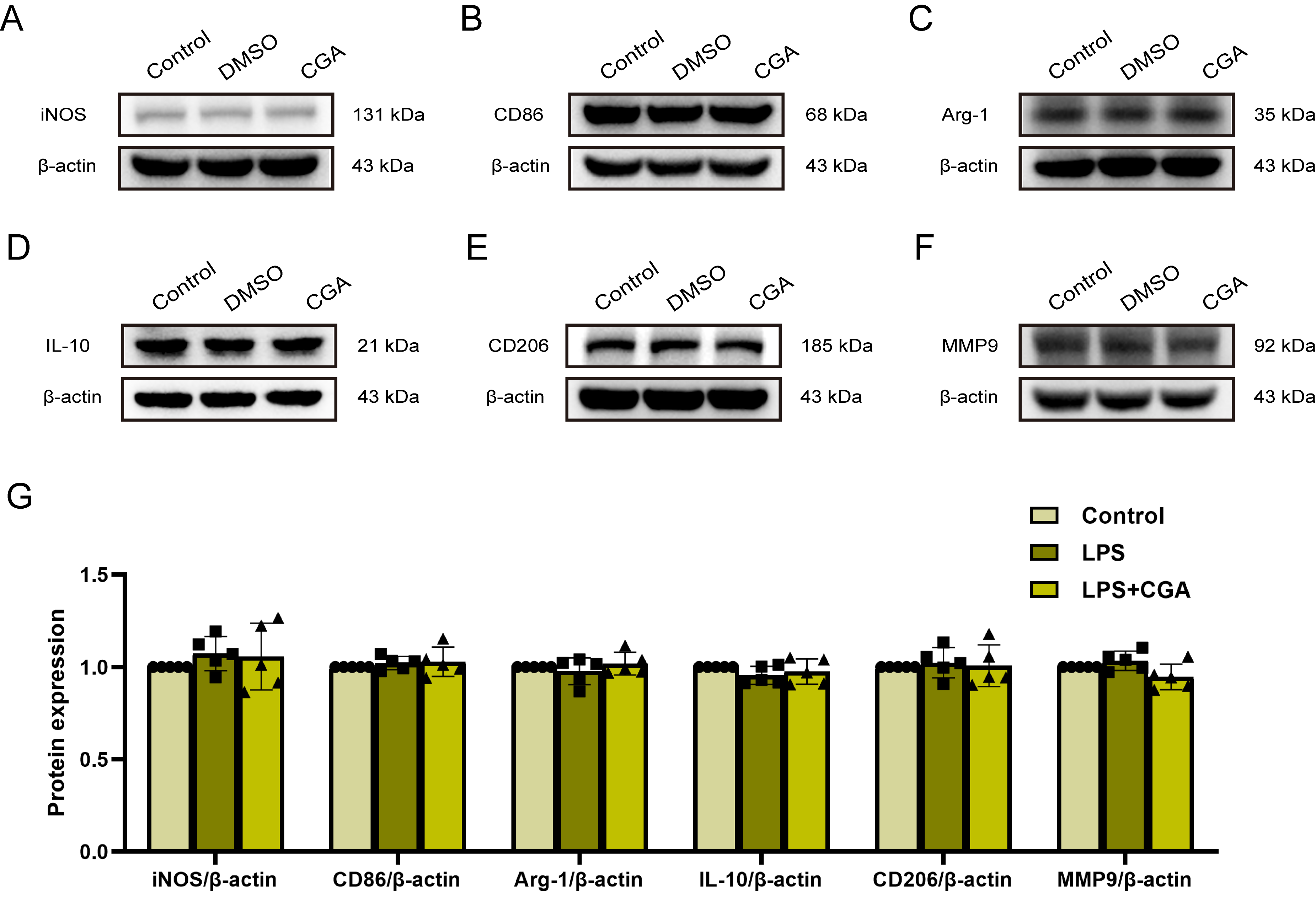


**Supplementary Figure 2.** Effect of DMSO and chlorogenic acid(100 μM) on BV-2 cells. (A-F) Expression of iNOS, CD86, Arg-1, IL-10, CD206 and MMP9. (G) Bar graph showed related protein expression analysis.
